# Pseudo-Subarachnoid Hemorrhage after Cardiac Arrest

**DOI:** 10.5811/cpcem.2017.10.35856

**Published:** 2018-01-09

**Authors:** Kraftin E. Schreyer, Krishna Surapaneni, Maura Sammon

**Affiliations:** * Lewis Katz School of Medicine at Temple University, Department of Emergency Medicine, Philadelphia, Pennsylvania; † Lewis Katz School of Medicine at Temple University, Department of Radiology, Philadelphia, Pennsylvania

## CASE PRESENTATION

A 24-year-old man presented after presumed atraumatic cardiac arrest. He had prolonged resuscitation that ultimately resulted in return of spontaneous circulation. A non-contrast computed tomography (CT) brain was immediately obtained. Comparison was made to the patient’s prior head CT (Image).

## DIAGNOSIS

*Pseudo-subarachnoid hemorrhage* (pseudo-SAH). The pseudo-SAH phenomenon can be seen with anoxic brain injury and many other causes of diffuse cerebral edema.^2^ In anoxic brain injury, the hyperdense appearance results from a combination of loss of gray-white differentiation, narrowing and effacement of the subarachnoid spaces, and corresponding engorgement of superficial pial veins.^3^,^4^

Although the CT mimics the appearance of SAH, as evidenced by apparent diffusely increased density of the basal cisterns and subarachnoid spaces, this is perceptually artifactual, as the attenuation values are lower than expected for acute blood products. The Hounsfield units (HU) in pseudo-SAH are generally 30–45 vs. 60–70 in true SAH.^1^ Additionally, true SAH will have higher attenuation values than that of the tentorium, a helpful differentiating feature. In this case, HU were 42 at the basal cisterns and 43 at the tentorium. Additional differentiating features are the diffuse loss of gray-white differentiation and effaced basal cisterns indicating diffuse cerebral edema.^1^,^2^

The prognosis is worse in patients with pseudo-SAH vs. SAH, likely because of underlying disease processes and decreased cerebral perfusion in the setting of elevated intracranial pressure.^1^ Pseudo-SAH must be included in the differential for a patient with this CT appearance, because it may facilitate end-of-life discussions regarding invasive procedures, transfers, and/or do-not-resuscitate status.

CPC-EM Capsule

What do we already know about this clinical entity?

Pseudo-subarachnoid hemorrhage (pseudo-SAH) can be seen with many causes of diffuse cerebral edema, including anoxic brain injury.

What is the major impact of the image(s)?

This CT, seen after cardiac arrest, may indicate diffuse anoxic injury, rather than acute subarachnoid bleed.

How might this improve emergency medicine practice?

Including pseudo-SAH in the differential may facilitate end-of-life discussions regarding invasive procedures, transfers, or do-not-resuscitate status.

## Figures and Tables

**Image:**
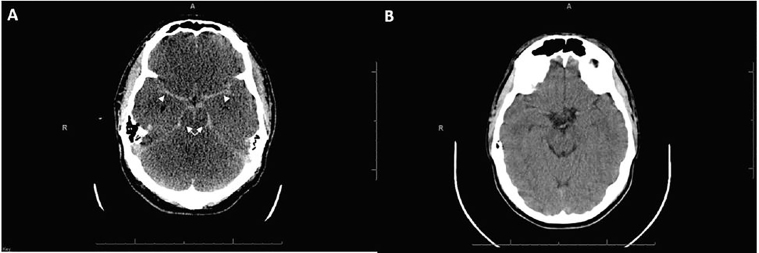
A) Post-resuscitation axial computed tomography (CT) of the brain demonstrating diffusely increased density of the basal cisterns (arrows) and subarachnoid spaces (arrowheads); and B) normal axial CT of the brain obtained on prior visit.
